# Ethanol affects behavior and HPA axis activity during development in zebrafish larvae

**DOI:** 10.1038/s41598-020-78573-y

**Published:** 2020-12-08

**Authors:** Wenxiao Du, Xiaoli Chen, Min Shi, Fuhua Bian, Zhenjun Zhao

**Affiliations:** 1 College of Life Science, Yantai University, Laishan District Spring Road No. 30, Yantai, 264005 Shandong People’s Republic of China; 2 The Fruit Trees Work Station of Penglai, Penglai Dengzhou Road, No. 67, Yantai, 265600 Shandong People’s Republic of China

**Keywords:** Biological techniques, Genetics, Molecular biology, Zoology

## Abstract

Recent studies have shown that long-term alcohol intake from food can lead to numerous mental disorders in humans, and the social and health effects of excessive intake of alcohol currently represent serious problems for governments and families worldwide. However, to date, it has not been determined how alcohol affects the hypothalamic–pituitary–adrenal (HPA) axis. The zebrafish offers a good model for studying the toxicology of food-grade ethanol. In the present study, using zebrafish larvae exposed to 1% ethanol, we performed zebrafish behavioral analysis. Samples were collected for enzyme-linked immunosorbent assay (ELISA) and quantitative real time-polymerase chain reaction (qRT-PCR) experiments, and statistical analysis was performed. We found that ethanol decreased the locomotor activity of zebrafish larvae, which showed a more intense reaction to external stimuli. Ethanol also increased the level of HPA axis hormones in zebrafish larvae, influenced the level of neurotransmitters, and altered the expression of key genes in neurotransmitter metabolism. Ethanol exposure affects zebrafish behavior, increases the level of HPA axis hormones in zebrafish larvae, affects the level of neurotransmitters, and affects the expression of key genes in dopamine and serotonin metabolism. These findings may help to elucidate the effects of ethanol on HPA axis activity.

## Introduction

Ethanol is a substance consumed daily by humans that can cause considerable harm to human health, especially in minors^1^. Indeed, the social and health effects of alcoholism are among the most serious problems faced by governments and families every year^2^. Recent studies have found that long-term consumption of ethanol can lead to numerous physical^2^, behavioral^3^, and mental disorders^4^. Furthermore, drinking during pregnancy can have a serious impact on fetal development, leading to fetal alcohol syndrome^5^. The main manifestations of this syndrome are skull deformity, cardiovascular dysplasia, and permanent nervous system damage^6^. Long-term drinking can also increase the risk of stroke, dementia, and hypertension, increase blood lipid concentration, and lead to a higher incidence of various cardiovascular and cerebrovascular diseases^7^. Because the liver and kidney are the primary organs of alcohol metabolism, acute alcoholism can lead to alcoholic liver and kidney diseases^5^. The most common pathological changes in alcoholic liver are a decrease in the rough endoplasmic reticulum, swelling and deformation of the mitochondria, and the occurrence of liver cirrhosis^8^. Epidemiological studies have also demonstrated that alcoholics are more likely to develop liver cancer. More recent studies have revealed that long-term drinking is associated with major mental disorders, such as depression^9^.

Numerous studies on alcohol toxicity have been based on the zebrafish model^10^. The zebrafish (*Danio rerio*), as a vertebrate, has a genome with a high degree of homology with that of humans, which makes it a valuable model for developmental, neurological, and toxicological research^11^. For example, Lockwood et al.^12^ found that embryonic exposure to ethanol increased the susceptibility of larval zebrafish to chemically induced seizures^11^. Similarly, Matsui^13^ found that high concentrations of ethanol can lead to abnormal development of the visual system, while even low concentrations of ethanol can lead to behavioral disturbances^12^. Carva et al.^14^ found that the learning ability of adult and juvenile zebrafish treated with ethanol decreased significantly^14^. Accordingly, zebrafish is a good model for studying the toxicology of ethanol. While ethanol has been observed to cause depressive phenotypes in behavioral experiments, the underlying mechanism of this effect has not been elucidated.

The hypothalamic–pituitary–adrenal (HPA) axis is a bodily system that involves the hypothalamus, pituitary gland, and adrenal gland and is activated in response to environmental stress^15^. Specifically, the hypothalamus secretes corticotropin releasing-hormone (CRH) which, in turn, acts on the pituitary gland to release adreno-cortico-tropic-hormone (ACTH). ACTH then acts on the adrenal gland to secrete glucocorticoids (GCs), which regulate HPA axis activity through glucocorticoid receptors (GR or nr3c1)^16^. Cortisol is the main glucocorticoid in zebrafish, while corticosterone is the main glucocorticoid in rodents^17^. The HPA axis is an important aspect of the neuroendocrine system, as it controls the stress response and regulates numerous physical activities ^16^. Numerous neurotransmitters are involved in regulating hypothalamic activity which, in turn, regulates the activity of the entire HPA axis, including specific changes in mood^18^. Clinical studies have shown that many patients with depression exhibit decreased GR expression and increased HPA axis activity^19^. On day 4 postfertilization (dpf), zebrafish show a fully functional HPA axis, and the HPA axis of zebrafish has remarkably similar functions. Indeed, zebrafish have been successfully utilized in studies on the HPA axis. Thus, zebrafish offer a good model for studying the function of the HPA axis^20^.

To determine whether the HPA axis is a target of ethanol, in this study, we exposed zebrafish larvae to 1% ethanol and examined their subsequent behavior, hormone levels, neurotransmitter levels, and gene expression levels. We attempted to determine the effects of ethanol as a stress factor on zebrafish behavior related to the HPA axis.

## Results

### Exposure to ethanol affects zebrafish behavior

**Figure 1:**
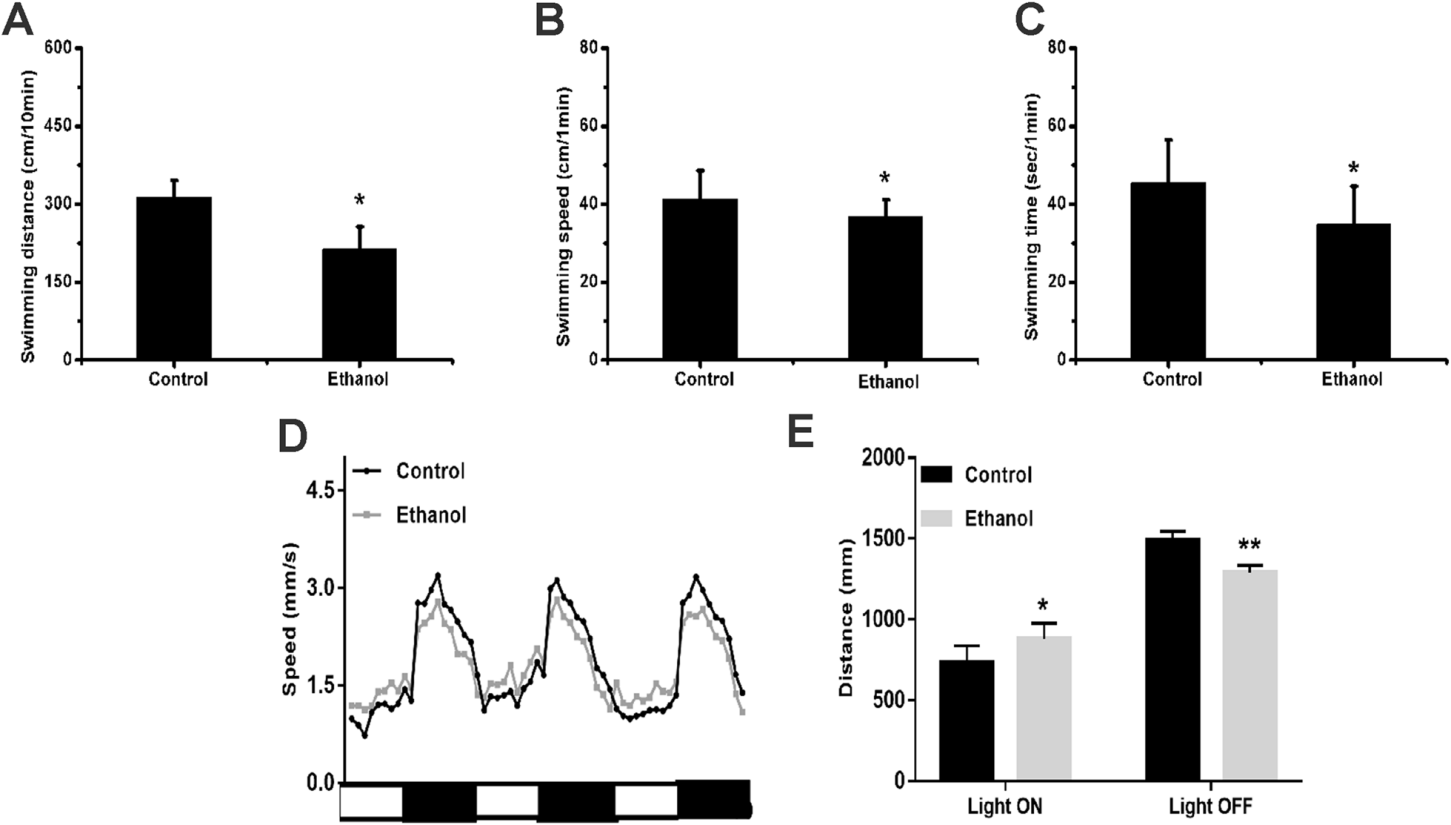
Effects of ethanol exposure on zebrafish locomotor activity. After exposure to alcohol, we investigated the behavior of 120 hpf zebrafish larvae. **(A)** Total swimming distance in 10 min. **(B)** Swimming speed in 1 min. **(C)** Swimming time in 1 min. **(D)** Swimming distance in LD time intervals. **(E)** Total swimming distance in LD time intervals. Each group, n = 24. Student’s t-test was conducted. *p < 0.05; **p < 0.01; ***p < 0.001.

To investigate whether ethanol affects the autonomous behavior of zebrafish, we selected 120 hpf control and ethanol-exposed zebrafish larvae for behavioral analysis. The results showed that under identical conditions, the swimming distance of zebrafish exposed to 1% ethanol was significantly shorter than that of the control fish (Fig. 1A), while the swimming speed per unit time was significantly lower (Fig. 1B). Furthermore, the average swimming time per unit time of the ethanol group was significantly lower than that of the control group (Fig. 1C). Taken together, these results suggest that exposure to ethanol decreases the locomotor activity of zebrafish larvae.

To test the exposed zebrafish’s ability to respond to external stimuli, we observed their responsiveness to alternating stimulation of light and dark (30 min of light and 30 min of darkness). Compared to the control group, the ethanol group showed significantly greater activity under light conditions (Fig. 1D,E) but a significantly reduced swimming distance under dark conditions (Fig. 1D,E). These findings indicate that the ethanol-exposed zebrafish had a more intense reaction to external stimuli.

### Exposure to ethanol affects the level of HPA axis hormones

**Figure 2:**
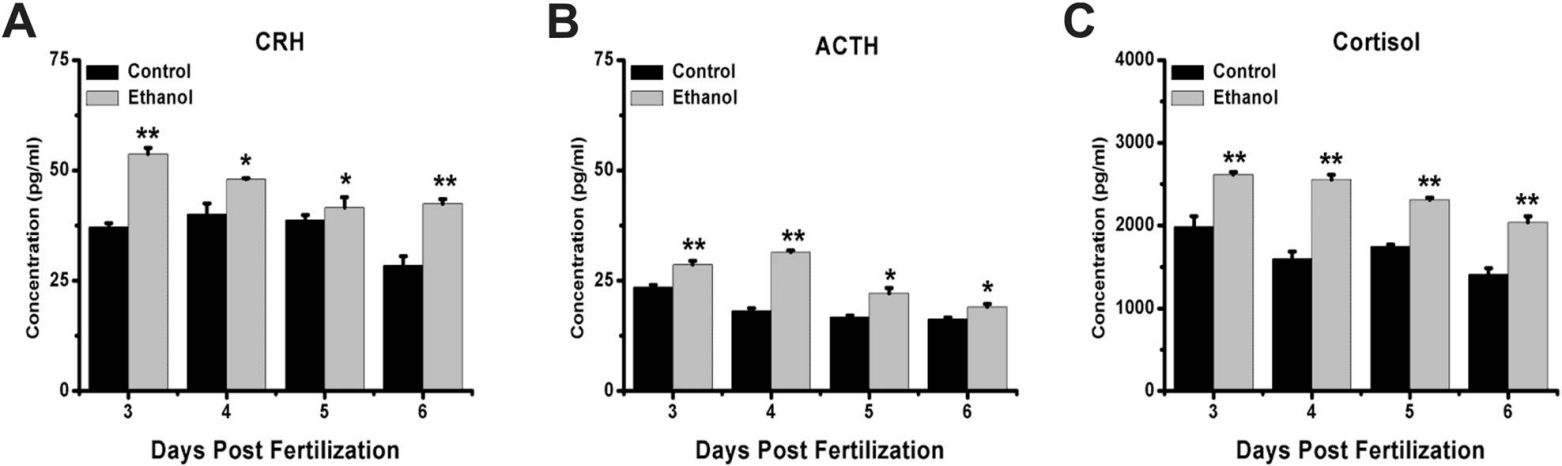
Effects of ethanol on HPA axis hormone levels in zebrafish larvae. Samples were taken at 3, 4, 5, and 6 dpf. **(A)** CRH levels from 3 to 6 dpf. **(B)** ACTH levels from 3 to 6 dpf. **(C)** Cortisol levels from 3 to 6 dpf. Three independent experiments were conducted. Student’s t-test was performed. *p < 0.05; **p < 0.01; ***p < 0.001.

To investigate whether ethanol exposure affects zebrafish HPA axis activity, we used ELISA to detect the levels of related hormones. The results showed that the level of CRH was significantly elevated after exposure to ethanol at 3, 4, 5, and 6 dpf (Fig. 2A), as was the level of ACTH (Fig. 2B) and the level of cortisol (Fig. 2C). Taken together, these results suggest that ethanol increases the level of HPA axis hormones in zebrafish larvae.

### Exposure to ethanol affects the expression of HPA axis genes

**Figure 3:**
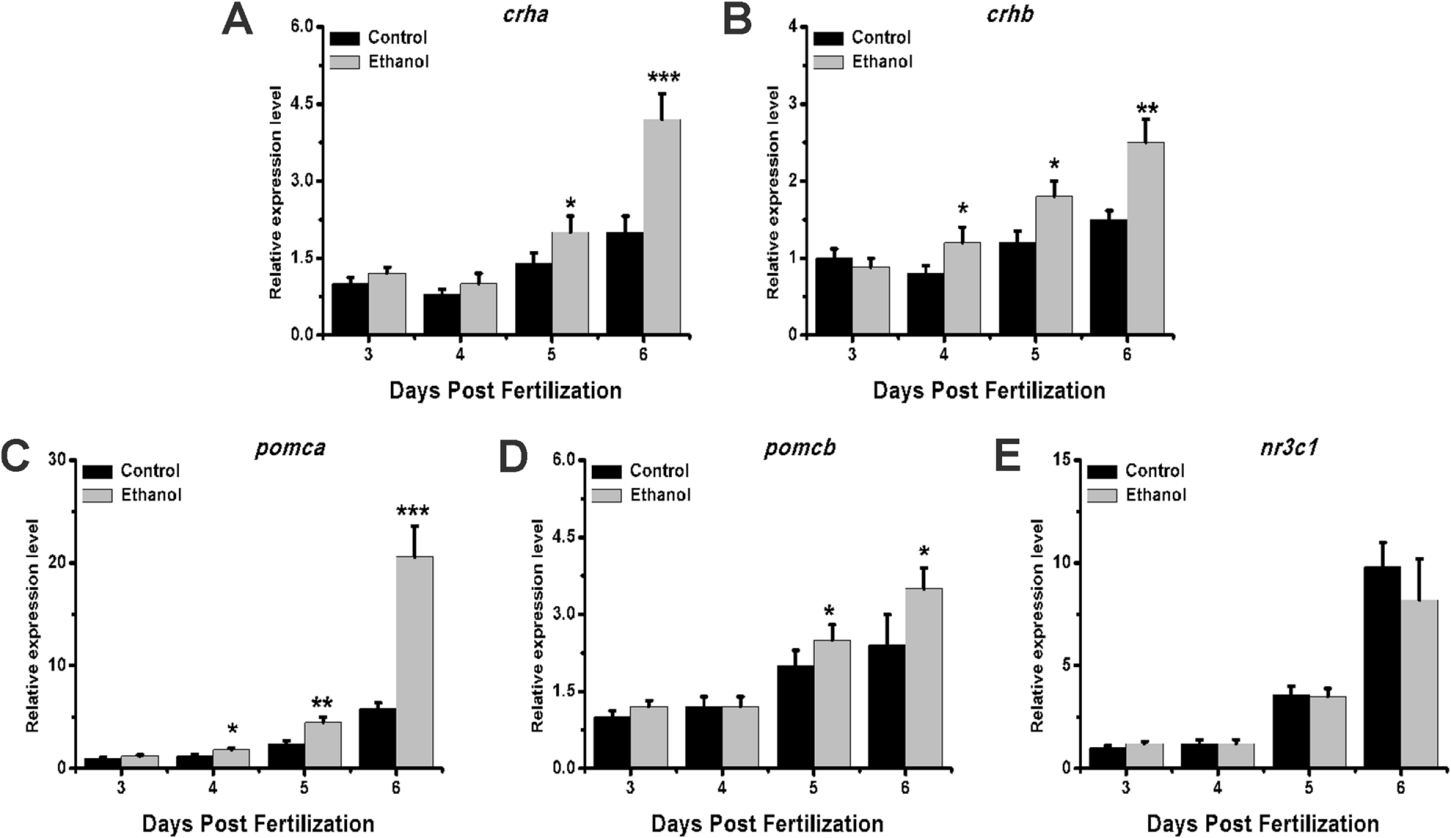
HPA axis gene expression patterns in zebrafish larvae after ethanol exposure. Samples were taken at 3, 4, 5, and 6 dpf. The expression patterns of **(A)** *crha*, **(B)** *crhb*, **(C)** *pomca*, **(D)** *pomcb*, and **(E)** *gr*. Three independent experiments were conducted. Student’s t-test was performed. *p < 0.05; **p < 0.01; ***p < 0.001.

To further investigate how ethanol affects HPA axis activity, we performed qRT-PCR analysis to investigate HPA axis gene expression. We found that the expression of *crha* exhibited no significant change at 3 and 4 dpf, but there was a significantly greater upregulation at 5 and 6 dpf in the ethanol group compared with the control group (Fig. 3A). Furthermore, the expression of the *crhb* gene showed a significant increase at 4, 5, and 6 dpf compared with the control group (Fig. 3B); there was no significant difference at 3 dpf (Fig. 3B). The expression of *pomca* and *pomcb* increased significantly with development time. Specifically, the expression of pomca showed clear upregulation at 4, 5, and 6 dpf, but there was no significant difference at 3 dpf (Fig. 3C). The expression of *pomcb* did not show a significant difference at 3 and 4 dpf, but it did show significant upregulation at 5 and 6 dpf in the ethanol group (Fig. 3D). The expression of *nr3c1* increased with the development time, although there was a slight decrease in expression at 6 dpf, and the ethanol group did not show significant differences from the control group (Fig. 3E). Taken together, these results indicate that the HPA axis gene expression patterns of zebrafish larvae were notably affected by exposure to ethanol.

### Exposure to ethanol affects the level of neurotransmitters

**Figure 4:**
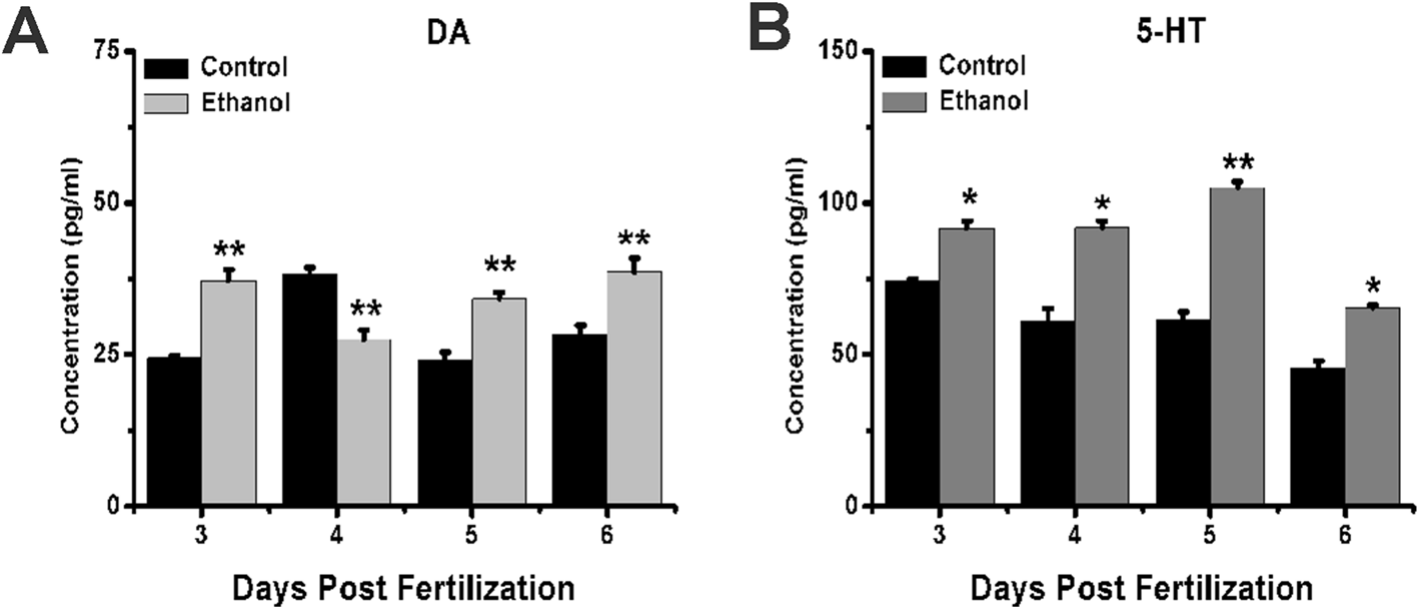
Level of neurotransmitters in zebrafish larvae after ethanol exposure. Samples were taken at 3, 4, 5, and 6 dpf. **(A)** Dopamine concentration from 3 to 6 dpf. **(B)** 5-HT concentration from 3 to 6 dpf. Three independent experiments were conducted. Student’s t-test was performed. *p < 0.05; **p < 0.01; ***p < 0.001.

Numerous monoamine neurotransmitters play important roles in the regulation of the HPA axis, particularly the regulation of an individual’s behavior and mood. To investigate whether ethanol exposure influences the level of neurotransmitters, particularly dopamine and serotonin, we used ELISA. The results showed that the level of dopamine showed a significantly stronger increase at 3, 5, and 6 dpf in the ethanol group than in the control group, although it decreased at 4 dpf (Fig. 4A). Serotonin, on the other hand, showed significantly stronger increases at all four time points in the ethanol group compared to the control group (Fig. 4B). These results indicate that exposure to ethanol influences the level of neurotransmitters.

### Exposure to ethanol affects the expression of key genes in dopamine and serotonin metabolism

**Figure 5:**
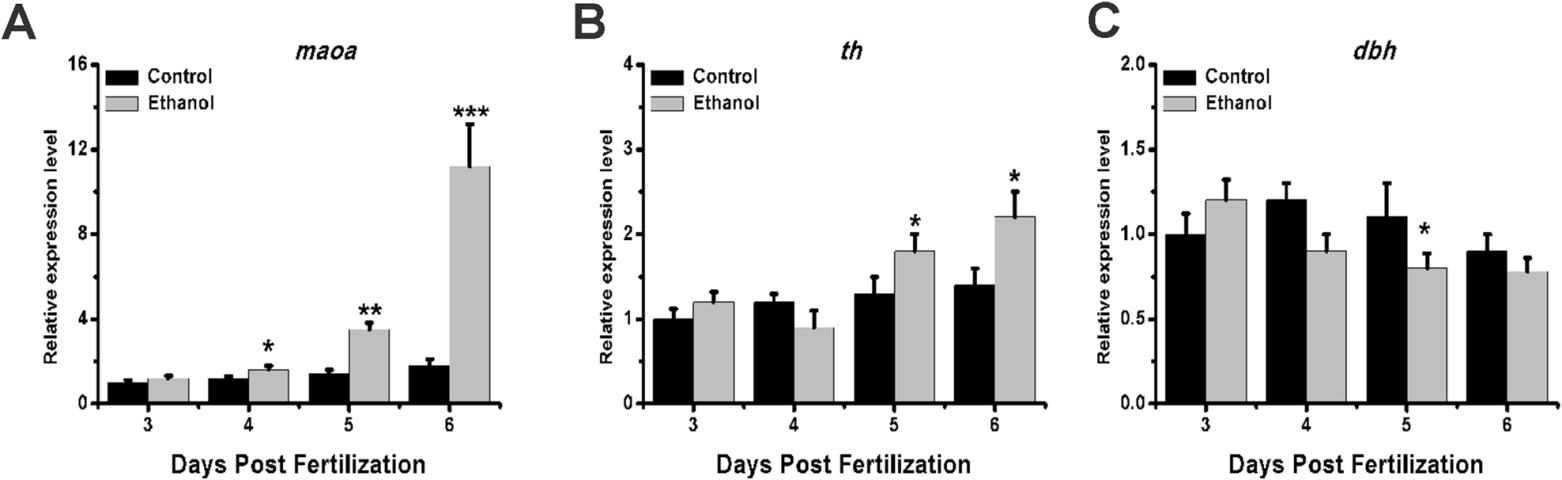
Neurotransmitter metabolism gene expression patterns in zebrafish larvae after ethanol exposure. Samples were taken at 3, 4, 5, and 6 dpf. The expression patterns of **(A)** *maoa*, **(B)** *th*, and **(C)** *dbh* were observed. Three independent experiments were conducted. Student’s t-test was performed. *p < 0.05; **p < 0.01; ***p < 0.001.

Key genes act as rate-limiting enzymes in the synthesis and metabolism of certain neurotransmitters. We selected three key genes that served as rate-limiting enzymes for the above two neurotransmitters and performed qRT-PCR analysis. The results showed that compared with the control group, the expression of monoamine oxidase-a(*maoa*) significantly increased at 4, 5, and 6 dpf in the ethanol group; however, there was no significant difference at 3 dpf (Fig. 5A). Furthermore, the expression of tyrosine hydroxylase(*th*) was significantly greater in the ethanol group at 5 and 6 dpf but showed no significant difference at 3 and 4 dpf (Fig. 5B). Finally, the expression of dopamine β-hydroxylase(*dbh*) was significantly downregulated at 5 dpf in the ethanol-treated group compared with the control group (Fig. 5C). Thus, exposure to ethanol affects the expression of key genes during neurotransmitter metabolism.

## Discussion

Zebrafish are a good model organism for studying neuroendocrine and behavioral effects^20^. There are numerous established behavioral and physiological tests and methods for generating genetic mutations in zebrafish, and numerous studies have utilized zebrafish to study ethanol toxicology^21^. In this study, we employed behavioral experiments, hormone measurements, and gene expression analyses to explore the effects of ethanol and found that ethanol affected zebrafish larvae activity, increased HPA axis activity, and disrupted neurotransmitter metabolism.

Exposure to 1% ethanol led to changes in the swimming behavior of zebrafish, including decreased swimming distance and speed. Furthermore, the light–dark alteration experiment showed that ethanol-exposed zebrafish showed significantly more activity in the light and lower activity under dark conditions than did the zebrafish in the control group. In the natural environment, sudden darkness is a dangerous environment for animals and can cause animals to increase their activity^22^. Thiago Acosta et al. reported that alcohol impairs predation risk response and communication in zebrafish^27^. This finding suggests that under light conditions, compared with the control zebrafish, 1% ethanol-treated zebrafish had significantly higher activity,while a significantly reduced swimming distance under dark conditions.

As noted above, the HPA axis is the primary system by which animals respond to changes in their external environment and stress, and the HPA axes of zebrafish and humans are highly similar^24^. Past studies have shown that the shallow water test leads to a clear increase in cortisol level and HPA activity^25^. Previous studies revealed that low concentrations of alcohol decrease cortisol production^26^, but our results show that 1% alcohol increases cortisol production^27^. In contrast to previous results, alcohol exposure reduces HPA axis activity^22–24^, and we think two reasons may explain this difference. First, for the exposure time, we exposed zebrafish larvae at 24 hpf; at this time, the HPA axis matured early, indicating that early-stage alcohol exposure can affect HPA axis activity. Second, in our experiment, ethanol exposure lasted for a long time, which was one of the reasons for the difference in results from the previous study.

GR is the core molecule in the HPA axis that regulates downstream gene expression. GR expression is usually downregulated in patients with depression^28^. In zebrafish, deletion of the *gr* gene can also lead to mood disorder^29^. In our study, we saw a trend to decrease in the expression of the gr gene. In addition, we found no difference in HPA axis activity at 3 and 4 dpf, possibly because the zebrafish larvae did not form a fully functional HPA axis before 4 dpf^30^. In addition, numerous compounds encountered during pregnancy can lead to epigenetic changes in gr^31^ which, along with development, have an effect on adolescents’ personality and mood^32^. Whether embryo or maternal alcohol exposure can lead to differences in the epigenetic regulation of zebrafish gr and mood changes in later developmental stages warrants further investigation.

The HPA axis is a neuroendocrine regulation axis linked to the level of neurotransmitters associated with the development of mental illness^33–35^, particularly dopamine^36^ and serotonin^33,35^. We found significant increases in the levels of both neurotransmitters after exposure to ethanol along with increased expression of the *maoa* and *th* genes and decreased expression of the *dbh* gene. The *th*^37^ is a rate-limiting enzyme in serotonin synthesis, *dbh*^38^ converts dopamine into norepinephrine, and maoa^34,36^ genes encode rate-limiting enzymes in dopamine metabolism. Thus, our results suggest that ethanol exposure increases the levels of dopamine and serotonin, perhaps partly due to decreased *dbh*. Previous studies have also found that in the hypothalamus, dopaminergic and serotonergic neurons can project to ACTH neurons to regulate the HPA axis^37^; however, the detailed mechanism of this projection has not been elucidated. Whether ethanol exposure leads to changes in neurotransmitters that, in turn, cause behavioral changes or whether ethanol exposure directly affects behavioral abnormalities caused by HPA axis activity is a matter for further research.

## Conclusion

Ethanol exposure was observed to reduce zebrafish locomotor activity, increase HPA axis activity, and lead to significant changes in the levels of dopamine and serotonin. These results provide a new understanding of the effects of ethanol on the HPA axis.

## Methods and materials

All experimental procedures were approved by the Yantai University Animal Care and Use Committee and were in accordance with governmental regulations of China.

### Fish maintenance

Adult zebrafish of the AB line were raised in a recirculating water system under a 14/10-h light/dark (L/D) cycle at 28 °C. The zebrafish were fed three times per day. To produce embryos, two male and two female adult zebrafish were paired in one 2-L tank in the evening, and spawning occurred the next day within 1 h after the lights were switched on. The embryos were placed in 10-cm Petri dishes with egg water containing methylene blue (0.3 ppm) and were raised in a light-controlled (14/10-h L/D) incubator at 28 °C.

### Ethanol treatment procedure

According to data obtained in a previous experiment^27^, 1% ethanol significantly alters zebrafish larvae behavior and gene expression; therefore, at 24 h postfertilization (hpf), 50 larvae were exposed to 25 ml 1% ethanol (directly dissolved in E3 solution) in each petri dish. A total of 500 embryos were treated. A control group consisting of sibling larvae raised in E3 solution (34.8 g NaCl/L; 1.6 g KCl/L; 5.8 g CaCl_2_·2H_2_O/L; 9.78 g MgCl_2_·6H_2_O/L) was also applied. Each treatment was repeated three times, and three independent experiments were performed. For each group, the ethanol solution was refreshed daily until the sampling stage was reached. At 3, 4, 5, and 6 dpf, 60 larval fish were collected for the enzyme-linked immunosorbent assay (ELISA), and 30 larval fish were collected for quantitative real time-polymerase chain reaction (qRT-PCR) experiments.

### Zebrafish behavioral analysis

Locomotor activity analysis was performed as described in a previous study with several modifications^38^. At 5 dpf, a single larva was placed in each well of a 48-well plate (24 control and 24 ethanol-exposed larvae), thereby enabling simultaneous tracking of the larvae. The locomotor activities of the larvae were monitored for 30 min using an automated video-tracking system (Videotrack, ViewPoint LifeSciences, Lyon, France), while their movement was recorded and analyzed using Zebralab 3.10 Software (Videotrack, ViewPoint Life Sciences, Lyon, France). The 48-well plates were placed inside the Zebrabox Observation Chamber, where they were exposed to continuous infrared light or 10 min light ON and 10 min light OFF. Instruments were placed in the chamber to maintain a constant temperature of 28.5 °C. The Videotrack quantization parameters were set as in the previous study^38^. The test was performed three times. Data were further analyzed using Microsoft Excel.

### RNA extraction and qRT-PCR

For the qRT-PCR analysis, total RNA was extracted using TRIzol Reagent (Invitrogen) according to the manufacturer’s instructions. Following DNase treatment, RNA (1 µg) was reverse-transcribed with oligo (dT) 18 primers and M-MLV reverse transcriptase. qRT-PCR analysis was then performed in the StepOne Real-Time PCR System using SYBR Premix Ex Taq (Takara Bio Inc., Dalian, Liaoning). At least three independent samples were examined in this analysis, with each being examined in triplicate. The mRNA level of the genes of interest was calculated using the 2−∆CT method and presented as relative (-fold) values of the control group after normalization to the β-actin mRNA levels.

The target gene levels were normalized to β-actin, and triplicates were run for each sample. In each reaction with the target gene primers, 1 µl of undiluted cDNA was used, whereas the cDNA used for β-actin amplification was diluted 1:10. The relative expression of the target genes was calculated using the 2(−ΔΔCT) method. The relative primers are listed in Table 1.

**Table 1:** The relative primers of genes.

Gene name	Forward primer	Reverse primer	Note
crha	CAGCAGACTCTCACCGACAA	CAGAGCTCCAGACGGAGAGT	qRT-PCR
crhb	CTCGCCACTTTTTGACATGA	GCTGCTCTCGATGGCTCTAC	qRT-PCR
pomca	GCTCAGTGTTGGGAAAATGC	GGTAGACGGGGGTTTCATCT	qRT-PCR
pomcb	GTGCAGATCGGACCAAGAAT	GCAAACCCAAGCTCAGACTC	qRT-PCR
nr3c1	GGCCAGTTTATGCTTTTCCA	TTGTGTGTGCCAGTCTTTCC	qRT-PCR
maoa	GAATCCTGTGGTCCTGGAAG	GAATGCGGTTTTGAGTTGGT	qRT-PCR
th	CAGCTCCACATCTTCCACAA	CGCATCCTCGATCAAACTCT	qRT-PCR
dbh	AAGAGCTCCATCATGGCATT	CTGCCTTCACTGTCACTCCA	qRT-PCR
β-actin	ATCACAGTTCCAGCCTATTTC	TGCCGTCTTCGATGGTCAG	qRT-PCR

### Enzyme-linked immunosorbent assay (ELISA)

Samples from the ethanol and control groups in the 3, 4, 5 and 6 dpf zebrafish were extracted. We analyzed HPA axis hormones and neurotransmitters using an ELISA kit obtained from Fanke Biotech (Shanghai, China) according to the manufacturer’s protocol.

### Statistical analyses

The data are presented as the mean ± SD. Statistical differences between two or more groups were determined using Student’s t-test and one-way analysis of variance (ANOVA) followed by the Student–Newman–Keuls test, respectively. All experiments were repeated three times, and the representative results are described in “Discussion”. A p-value < 0.05 was considered to indicate significance.
